# Effectiveness of simulation-based interventions on empathy enhancement among nursing students: a systematic literature review and meta-analysis

**DOI:** 10.1186/s12912-024-01944-7

**Published:** 2024-05-11

**Authors:** Mi-Kyoung Cho, Mi Young Kim

**Affiliations:** 1 Department of Nursing Science, Chungbuk National University, 1 Chungdae-ro, Seowon-gu, Cheongju, Korea; 2 College of Nursing, Hanyang University, 222 Wangsimni-ro, Seongdong-gu, Seoul, South Korea

**Keywords:** Simulation-based interventions, Empathy, Nursing students, meta-analysis

## Abstract

**Background:**

This study aimed to secure and analyze evidence regarding the enhancement of nursing students’ empathy through simulation-based interventions. It comprehensively analyzed self-reported emotions and reactions as primary outcomes, along with the results reported by nursing students who experienced simulation-based interventions, including empathy.

**Methods:**

This systematic literature review and meta-analysis investigated the effects of simulation-based interventions on enhancing empathy among nursing students. The Preferred Reporting Items for Systematic Reviews and Meta-Analyses guidelines were used for the systematic review and meta-analysis. The following details were considered: population, nursing students; intervention, simulation-based interventions targeting empathy enhancement; comparators, control groups without intervention or those undergoing general non-simulation-based classes; and outcomes, self-reported empathy.

**Results:**

In the systematic review of 28 studies, it was found that the use of simulation-based interventions among nursing students led to an increase in empathy, albeit with a small effect size. This was demonstrated through a pooled, random-effects meta-analysis, yielding an effect size (Hedge’s g) of 0.35 (95% CI: 0.14, 0.57, *p* = 0.001). The results of meta-regression and subgroup analysis significantly increased in empathy for studies published after 2019 (Hedge’s g = 0.52, 95% CI: 0.31 to 0.73, *p* < 0.001), quasi-experimental research design (Hedge’s g = 0.51, 95% CI: 0.27 to 0.74, *p* < 0.001), more than 60 participants (Hedge’s g = 0.31, 95% CI: 0.02 to 0.59, *p* = 0.034), and simulation-based interventions in nursing education (Hedge’s g = 0.43, 95% CI: 0.22 to 0.65, *p* < 0.001).

**Conclusions:**

Considering factors such as variations in sample size, research approaches, and the effects of independent studies on empathy, this systematic literature review and meta-analysis suggests that simulation-based education can significantly improve nursing students’ overall empathy skills.

**Supplementary Information:**

The online version contains supplementary material available at 10.1186/s12912-024-01944-7.

## Background

In modern society, concerns are growing regarding empathy deficits, which lead to issues such as indifference and apathy in workplace relationships—aggravating even in common social situations [1]. Empathy is a complex concept comprising an affective component of feeling and recognizing emotions from others’ perspectives and a cognitive component of understanding others’ emotions [2]. Highly empathetic professionals in health-related fields foster a high level of communication with patients, leading to positive outcomes in patient care, such as better self-care, higher patient satisfaction, and faster recovery times [3, 4]. Although empathy is essential for healthcare workers, studies have demonstrated that it is not taught sufficiently during training in numerous fields, including medicine, nursing, dentistry, and pharmacy [5, 6].

Empathy plays a crucial role in healthcare, as evidenced by its strong correlation with the quality of care provided to patients. When patients perceive that nurses empathize with them, they tend to feel they are receiving care tailored to their needs [7]. Therefore, improving empathy is necessary for enhancing the quality of nursing care. Efforts have been made to develop programs that foster empathy through education and training [8]. Adequate levels of empathy are essential for nursing students as they are future nurses. However, research has indicated that nursing students have lower levels of empathy than other healthcare workers do [9, 10].

Empathy is defined as the ability to place oneself in the same position as another person and to understand and accept their position and perspective [11]. Training that enhances empathy includes simulation-based learning that recreates realistic clinical situations [12]. Additionally, healthcare can be simulated in various ways, including virtual patients, manikins, role-playing, gaming, and simulating hypothetical or disease situations [13]. Simulations in healthcare most often allow students to function in the role for which they are training, though there is evidence students’ empathy increases when they function in the role of patients because they are encouraged to understand patients’ perspectives, emotions, and experiences [14]. Whether students function in professional or patient roles during simulation, post-simulation debriefing helps students translate their simulation experiences. Post-simulation debriefing sessions have been shown to help students learn how to translate their simulation experiences into appropriate empathetic behaviors and attitudes toward patients in the real world [14]. Previous systematic reviews have included studies focusing on specific simulation methods, such as role-play or virtually simulated patients, dementia-specific virtual reality scenarios, clinical simulations with dramatization, and simulation equipment for older-adult-specific scenarios [15]. Since its emergence, improving empathy in healthcare has been the subject of several studies and meta-analyses [16]. Through a meta-analysis and effectiveness evaluation study on various simulation-based programs aimed at nursing students, both future and current medical professionals, we investigated the elements of simulation that contribute to empathy enhancement. Our study identified key elements crucial for designing effective simulation education programs, which can be reflected upon in practice. By analyzing the components of simulation-based education that impact empathy enhancement, we can identify crucial elements to enhance empathy when implementing this approach.

Simulation is becoming more prevalent as an educational approach for instilling empathy in pre-service health professional students [17]. As these various forms of simulation are applied to improve empathy, a systematic review and analysis of nursing students are needed to determine their effectiveness and the factors that should be considered. Systematic reviews and meta-analyses can consider differences in sample sizes, variations in research approaches, and the effects of interventions in independent studies while integrating the results of the included studies. Therefore, a systematic review and meta-analysis will enable an assessment of the overall effectiveness of simulation-based education in improving empathy among nursing students. This study aims to provide a foundation for simulation-based interventions by conducting a systematic literature review and meta-analysis to examine their effectiveness in improving empathy among nursing students.

## Methods

### Study design

This systematic literature review and meta-analysis followed the Population, Intervention, Comparison, Outcome, and Study Design (PICO-SD) framework to determine the effectiveness of simulation-based interventions in improving empathy among nursing students.

### Eligibility criteria and outcome variables

This study was conducted according to the Preferred Reporting Items for Systematic Reviews and Meta-Analyses (PRISMA) guidelines [18]. This was prepared by referring to the PRISMA 2020 checklist (https://prisma-statement.org/PRISMAStatement/Checklist.aspx, accessed May 16, 2023). In line with this study’s purpose, a systematic literature search was conducted. The inclusion criteria were as follows: the study population (P) included nursing students who received simulation training; the intervention (I) included nursing education using simulation to promote empathy; the control I group comprised those who did not receive the simulation intervention as a comparison group; and for outcomes (O), the primary outcome was empathy, while the secondary outcomes wereempathic communication, interpersonal relationships, and competency. The first post-intervention value was used to calculate the effect size. The study design (SD) involved randomized controlled trials (RCTs) and quasi-experimental studies that included manuscripts published in English or Korean from May 1971 to April 2023. Only studies that reported means, standard deviations, and concrete sample sizes were included to merge the effect sizes for the primary and secondary outcomes. The exclusion criteria were as follows: studies that included students other than nursing students, interventions that were not simulations, measured variables that were not graphically represented such that effect sizes could not be merged, studies that only presented p-values or the number of participants in each group, studies with mean and standard deviation not available, and duplicate studies. Quasi-experimental studies with a single-group pretest-posttest design were excluded.

### Search strategies

Data were retrieved from eight electronic databases or e-journals, specifically PubMed, Cochrane, EMBASE, CINAHL, World of Science, SCOPUS, PQDT, and Research Information Sharing Service (RISS), for articles published in English and Korean from May 1971 to April 2023. The search protocol was registered in the PROSPERO International Prospective Register of Systematic Reviews (registration no. CRD42023423747, available at https://www.crd.york.ac.uk/prospero) on May 16, 2023. The search formula used was Medical Subject Headings (MeSH) and text words from titles and abstracts, and the search was conducted from April 24, 2023, to June 3, 2023. The terms used in the search were (“Simulation Training”[MeSH Terms] OR “simulate*”[All Fields]) OR (“psychodrama”[MeSH Terms] OR “psychodrama*”[All Fields] OR “role-play*”[All Fields]) for interventions, and (“Empathy”[MeSH Terms] OR “empath*”[All Fields] OR “Emotional Intelligence”[MeSH Terms] OR “Emotional Intelligence”[All Fields]) for results. The data collection process for the articles included in the analysis was based on a systematic review. A literature search was conducted by two authors (MYK and MKC) with the guidance of a meta-analysis expert.

### Quality assessment

The quality of the selected articles was independently assessed by two authors (MYK and MKC) using the Joanna Briggs Institute (JBI) checklist (Checklist for Randomized Controlled Trials, Checklist for Quasi-Experimental Studies [19–21]. In the initial quality assessment, no discrepancies were observed across most items. However, divergence arose regarding the clarity of blinding of outcome assessors to study participants. Upon thorough discussion, we agreed that a score would be assigned only if the methodology section of a study explicitly stated that outcome assessors were blinded to treatment assignment. The JBI RCT Checklist comprises the following 13 items: randomization, allocation concealment, pre-homogeneity verification, blinding (participants, interventors, and assessors), identical conditions other than experimental treatment, description of dropouts, analysis based on randomization, equivalence of outcome measures, appropriateness of outcome variable measures and statistical analysis methods, and appropriateness of the study design [19]. The JBI Quasi-Experimental Studies Checklist comprises the following nine items: certainty of cause and effect, pre-homogeneity verification, exposure to the same environment outside of the intervention, presence or absence of a control group, pre- and post-intervention effect measures, description of dropouts, equivalence of outcome measures, appropriateness of outcome variable measures, and statistical analysis methods [20]. The checklist scored “yes” as 1 and “unclear,” “no,” and “not applicable” as 0 for each item. Discrepancies in the quality assessment of the studies were resolved through consultation with a meta-analysis expert and discussions between the two authors (MYK and MKC) (Table 1).

**Table 1:** Quality assessment of the included studies

**Joanna Briggs Institute of Critical Appraisal Tools Checklist for Checklist for Randomized Controlled Trials**														**Total score**	**Range**	
**Study ID**	**Q1**	**Q2**	**Q3**	**Q4**	**Q5**	**Q6**	**Q7**	**Q8**	**Q9**	**Q10**	**Q11**	**Q12**	**Q13**			
3	1	0	1	0	0	1	0	1	1	1	1	1	1	9		
7	0	0	1	0	0	1	0	1	1	1	1	0	1	7		
8	1	0	1	0	0	1	0	1	1	1	1	1	1	9		
11	1	0	1	1	0	1	0	1	1	1	1	0	1	9		
12	0	0	1	0	0	1	0	1	1	1	1	0	1	7		
13	1	0	1	0	0	1	0	1	1	1	1	0	1	8		
14	1	0	1	0	0	1	0	1	1	1	1	0	1	8		
16	1	0	1	0	0	1	0	1	1	1	1	0	1	8		
20	1	0	1	0	0	1	0	1	1	1	1	1	1	9		
21	1	0	1	0	0	1	0	1	1	1	1	0	1	8		
23	1	0	1	0	0	1	0	1	1	1	1	0	1	8		
Total	9	0	11	1	0	11	0	11	11	11	11	3	11	8.18	7–9	
**Joanna Briggs Institute of Critical Appraisal Tools Checklist for Quasi-experimental study**														**Total score**	**Range**	
**Study ID**	**Q1**	**Q2**	**Q3**	**Q4**	**Q5**	**Q6**	**Q7**	**Q8**	**Q9**							
1	1	1	1	1	1	0	1	1	1					8		
2	1	0	1	1	1	0	1	1	0					6		
4	1	1	1	1	1	1	1	1	1					9		
5	1	1	1	1	1	1	1	1	1					9		
6	1	0	1	1	1	0	1	1	0					6		
9	1	1	1	1	1	0	1	1	1					8		
10	1	1	1	1	1	1	1	1	1					9		
15	1	1	1	1	1	0	1	1	1					8		
17	1	1	1	1	1	0	1	1	1					8		
18	1	0	1	1	1	0	1	1	1					7		
19	1	1	1	1	1	1	1	1	1					9		
22	1	0	1	1	1	1	1	1	0					7		
24	1	1	1	1	1	1	1	1	1					9		
25	1	1	1	1	1	1	1	1	1					9		
Total	14	10	14	14	14	7	14	14	11					8.00	6–9	

### Selection process

The two authors (MYK and MKC) shared the search formula, searched for data independently, and shared the bibliographic information of the articles retrieved from domestic and foreign core electronic databases and journals in an Excel file. Duplicate articles were removed by sorting by title and author using the Microsoft Excel filtering function. Based on this search strategy, relevant articles were identified through titles and abstracts, after which the full texts of the selected articles were reviewed.

### Data analysis and statistical methods

The article characteristics were presented as frequencies, means, and standard deviations, and statistical analyses of effect size pooling methods were performed Z-test and p-value using MIX 2.0 Pro Ver. 2.0.1.6 (BiostatXL, Mountain View, CA, USA). As the effect sizes were continuous variables, and the number of participants in each study was small, Hedge’s g, 95% confidence intervals (CI), and the weight of each effect size were obtained using the inverse of variance [22]. The overall effect (Hedges’ g) was calculated using a pooled, random-effects model to account for between-participant variations in individual studies and heterogeneity among studies. The effect sizes indicated by Hedge’s g values of 0.15, 0.40, and 0.75 were classified as small, medium, and large effects, respectively [23]. The studies’ heterogeneity was assessed by calculating Higgin’s I^2^ value, which represented the true variance or variance ratio across studies to the total observed variance. It was interpreted as heterogeneous if I^2^ was greater than 50%. Subgroup and meta-regression analyses were performed to identify the sources of heterogeneity. Publication bias in the selected studies was tested using funnel plots, Begg’s test, Egger’s regression test, and the trim-and-fill method with a correction for Hedge’s g [24].

## Results

### Study selection

**Fig. 1:**
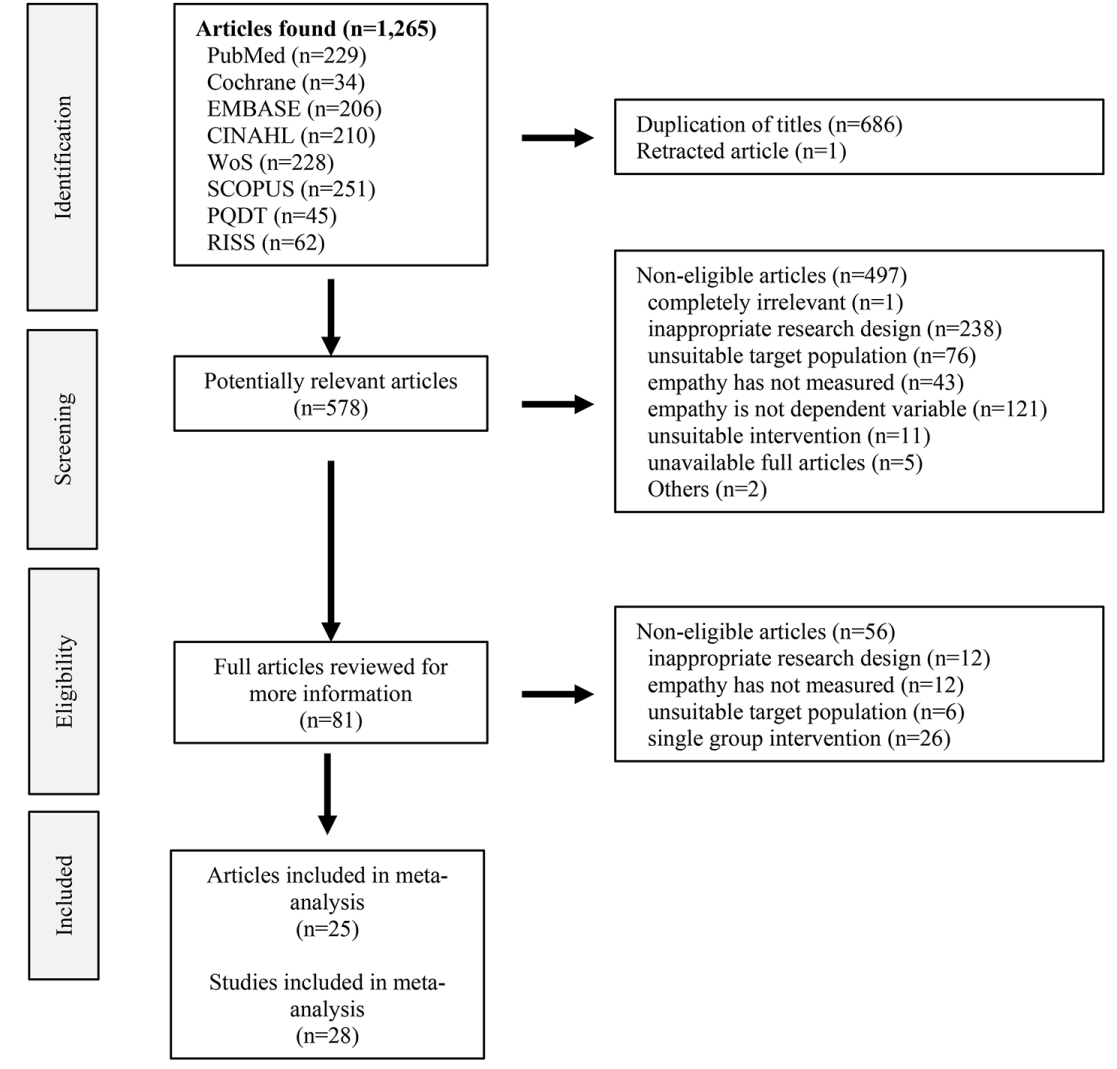
PRISMA flow diagram

This study followed the PRISMA guidelines during the study selection process, as illustrated in Fig. 1. A total of 1,265 articles were retrieved from each database in Step 1. Furthermore, 578 articles were extracted by excluding duplicate studies (686) and one retracted article in Step 2, and 81 articles were extracted by excluding studies that did not fulfill the inclusion and exclusion criteria in Step 3. Finally, after a thorough review and full-text reading, 25 articles meeting our search criteria were identified for inclusion. Notably, Layton’s (1979) study was distinguished by its comparison of experimental and control groups across four distinct simulation interventions. Given the unique structure of this study, each simulation intervention was treated as a separate unit of analysis, thereby extending the total number of analyzed studies to 28. In this study, the participants of the studies included in the meta-analysis were undergraduate nursing students, and a total of 2,598 participated. The data extraction form was compiled by extracting the author, year of publication, presence or absence of IRB, number of participants, research design, experimental group’s intervention type, intervention session, session time, control group’s intervention, post-test measurement time, delayed measurement, and outcome variables. The primary variable, empathy score, and the secondary variables, empathic communication, interpersonal relationships, and competency were coded as the mean, standard deviation, and number of samples of the first post-test or the difference value of the post-pretest for both the experimental and control groups after the intervention.

### Study characteristics

The analysis included 28 studies, with 15 published in 2019 or later, 23 with IRB reviews before the study. The research design for simulation-based interventions included 14 RCTs: 13 with 60 or more participants, 13 simulated-based learning, 15 role-plays, and 21 studies with usual or no interventions for the control group. Simulation-based learning encompasses a variety of structured activities designed to mirror real or potential scenarios in educational settings, facilitating practice and skill development. These activities enable participants to augment their understanding, expertise, and mindset, while also providing opportunities to analyze and address realistic situations within a simulated environment [25]. Role-playing entails the enactment of specific roles within defined contexts. For instance, it may encompass a situated teaching program where patients portray themselves and articulate their experiences within a psychiatric nursing practice setting, or a role-playing training regimen conducted within an operating room situation. The intervention time or session was more than 1 h, the outcome was measured immediately after the intervention, the outcome was followed up, pre-briefing was conducted, and debriefing was conducted in study ID: 24, 11, 12, 26, 10, 6, and 14 studies. The majority of the control group adheres to a Traditional curriculum. This curriculum typically includes conventional empathic skill training through lectures, seminars, individual presentations at meetings, discussions, and similar formats. In contrast, for the experimental group, simulation involves a sequence of processes (such as orientation, pre-briefing, SP simulation performance, debriefing, and feedback). Typically, this process occurs once rather than being repeated. The impact is evaluated following the completion of this singular series of processes. The predominant empathy scale utilized was The Jefferson Scale of Empathy-Health Profession-Student (JSE-HP-S), with various other assessment tools also employed to measure empathy.

The primary outcome was empathy, which was assessed in all 28 studies. Empathic communication, interpersonal relationships, and competency were measured in study ID: 5, 6, and 9 studies, respectively (Appendix 1). When the sample size is small, Cohen’s d may exaggerate the effect size of an individual study. Therefore, the adjusted effect size, referred to as Hedge’s g [25], was provided along with 95% Confidence Intervals. Hedge’s g was calculated by entering the mean, standard deviation, and number of samples of each study’s experimental and control groups into the Mix Pro 2.0 program.

### Risk of bias in studies

The average quality assessment score for RCTs was 8.18 (SD 0.75, range: 7–9), and the average quality assessment score for quasi-experimental studies was 8.00 (SD 1.11, range: 6–9). Among the internal validity assessment items for the RCT studies, “Q2. Was the allocation to treatment groups concealed?” for bias related to selection and allocation, and “Q5. Were those delivering the treatment blinded to the treatment assignment?” for bias related to administration of intervention or exposure, and “Were outcome assessors blind to treatment assignment?” for bias related to the assessment, detection, and measurement of the outcome were not reported in any study. Furthermore, “Q4. Were participants blinded to the treatment assignments?” was reported in only one study, and “Q12. Was an appropriate statistical analysis used?” was used to measure the validity of the statistical conclusions in three studies. Most items (Q1-5, Q7-9) that assess the quality of quasi-experimental studies have been reported. “Q6. Was the follow-up complete, and if not, were the differences between groups in terms of their follow-up adequately described and analyzed?” were reported in only seven studies (Table 1).

### Effect of simulation-based intervention on empathy

**Fig. 2:**
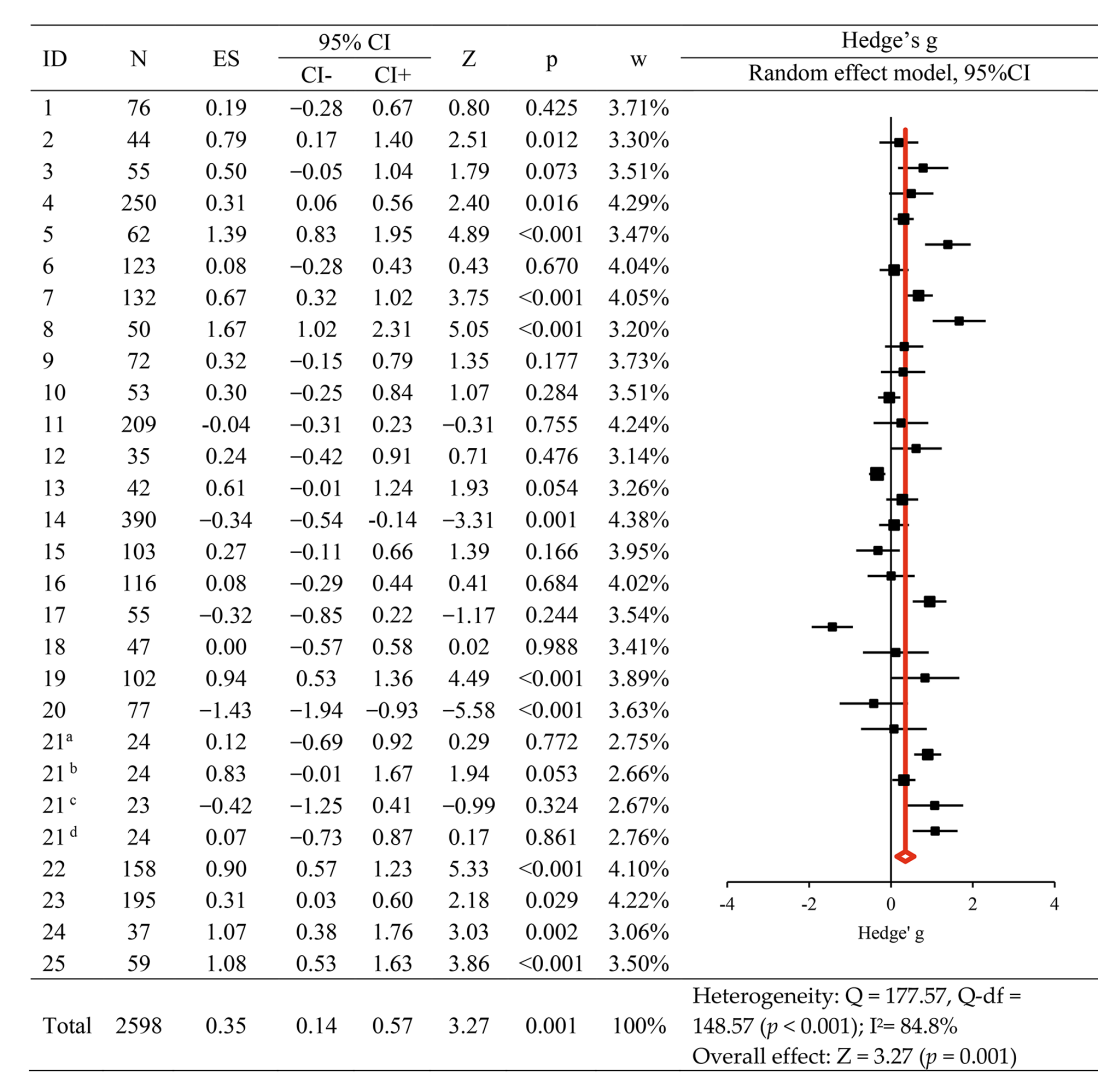
The effect of simulation-based intervention on empathy. Notes. ES: Effect size; CI: Confidence interval. Superscripts a, b, c, and d were Layton’s (1979) study divided by intervention

Layton’s (1979) study was distinguished by its comparison of experimental and control groups across four distinct simulation interventions. Each simulation intervention was treated as a separate unit of analysis, thereby extending the total number of analyzed studies to 28. The effect sizes were pooled using a random-effects model and presented as Hedge’s g, 95% CI, weight, and a synthesis forest plot (Fig. 2). Using a simulation-based intervention among nursing students significantly increased empathy, with a total effect size of Hedge’s g = 0.35, which was a small effect based on Brydges’ criteria for interpreting effect sizes. The effect sizes indicated by Hedge’s g values of 0.15, 0.40, and 0.75 were classified as small, medium, and large effects, respectively [26]. The heterogeneity test revealed a Higgins I^2^ value of 84.8%, indicating a high degree of heterogeneity among merged studies. Therefore, subgroup and meta-regression analyses were conducted for exploratory and descriptive heterogeneity analyses.

In the subgroup analyses, a significant increase in empathy was reported in the studies published after 2019 (Hedge’s g = 0.52, 95% CI:0.31, 0.73), IRB-approved studies (Hedge’s g = 0.39, 95% CI:0.15, 0.62), quasi-experimental studies (Hedge’s g = 0.51, 95% CI:0.27, 0.74), simulation-based interventions (Hedge’s g = 0.43, 95% CI:0.22, 0.65), and studies with no control group intervention or with usual interventions (Hedge’s g = 0.30, 95% CI:0.08, 0.53). The same was reported in studies with the intervention time per session not reported or less than 1 h (Hedge’s g = 0.42, 95% CI:0.20, 0.63), studies measuring the outcome right after the intervention (Hedge’s g = 0.38, 95% CI:0.16, 0.60), studies adopting no follow-up measurements for verifying the intervention’s long-term effects (Hedge’s g = 0.45, 95% CI:0.22, 0.68), and studies performing debriefing after simulation (Hedge’s g = 0.48, 95% CI:0.18, 0.78), compared to the studies that did not. Additionally, the effect sizes for the number of participants, pre-briefing, and quality assessment score were statistically significant (Table 2).

**Table 2:** Subgroup analysis of Empathy by Study Characteristics

Variables	Category		K	Study ID	*N*	Hedge’s g	95% CI		Z	*p*
							Lower limit	Upper limit		
Year		< 2019	13	2, 6, 8, 13, 14, 15, 17, 18, 20, 21^a^, 21^b^, 21^c^, 21^d^	987	0.13	−0.24	0.50	0.67	0.504
		≥ 2019	15	1, 3, 4, 5, 7, 9, 10, 11, 12, 16, 19, 22, 23, 24, 25	1611	0.52	0.31	0.73	4.93	< 0.001
		2	7	2, 5, 12, 13, 15, 16, 25	461	0.62	0.24	0.99	3.23	0.001
IRB		No	5	3, 11, 14, 16, 25	829	0.20	−0.21	0.61	0.94	0.346
		Yes	23	1, 2, 4, 5, 6, 7, 8, 9, 10, 12, 13, 15, 17, 18, 19, 20, 21^a^, 21^b^, 21^c^, 21^d^, 22, 23, 24	1769	0.39	0.15	0.62	3.24	0.001
Research design		Quasi-E	14	1, 2, 4, 5, 6, 9, 10, 15, 17, 18, 19, 22, 24, 25	1241	0.51	0.27	0.74	4.19	< 0.001
		RCT	14	3, 7, 8, 11, 12, 13, 14, 16, 20, 21^a^, 21^b^, 21^c^, 21^d^, 23	1357	0.19	−0.14	0.51	1.13	0.258
Participants		< 60	15	2, 3, 8, 10, 12, 13, 14, 17, 18, 21^a^, 21^b^, 21^c^, 21^d^, 24, 25	923	0.41	0.06	0.75	2.33	0.020
		≥ 60	13	1, 4, 5, 6, 7, 9, 11, 15, 16, 19, 20, 22, 23	1675	0.31	0.02	0.59	2.12	0.034
Intervention type		Simulation	13	1, 2, 3, 4, 6, 7, 8, 10, 11, 13, 18, 23, 24	1313	0.43	0.22	0.65	4.01	< 0.001
		Role-play	15	5, 9, 12, 14, 15, 16, 17, 19, 20, 21^a^, 21^b^, 21^c^, 21^d^, 22, 25	1285	0.25	−0.12	0.62	1.34	0.181
Control group intervention		No or usual	21	1, 3, 4, 6, 7, 8, 9, 10, 11, 15, 16, 17, 20, 21^a^, 21^b^, 21^c^, 21^d^, 22, 23, 24, 25	1876	0.30	0.08	0.53	2.60	0.009
		Comparison	7	2, 5, 12, 13, 14, 18, 19	722	0.51	−0.06	1.08	1.76	0.078
Intervention duration		Not reported or < 4weeks	17	1, 6, 8, 10, 12, 14, 16, 18, 19, 20, 21^a^, 21^b^, 21^c^, 21^d^, 22, 23, 25	1537	0.27	−0.05	0.58	1.67	0.095
		≥ 4weeks	11	2, 3, 4, 5, 7, 9, 11, 13, 15, 17, 24	1061	0.47	0.21	0.72	3.56	< 0.001
Intervention time/session		Not reported or < 1 h	16	1, 2, 6, 7, 8, 9, 10, 11, 12, 13, 21^a^, 21^b^, 21^c^, 21^d^, 22, 23	1245	0.42	0.20	0.63	3.74	< 0.001
		≥ 1 h	12	3, 4, 5, 14, 15, 16, 17, 18, 19, 20, 24, 25	1353	0.28	−0.10	0.66	1.44	0.150
Outcome measurement time		Immediately	26	1, 2, 3, 4, 5, 6, 7, 8, 9, 11, 12, 13, 14, 15, 16, 18, 19, 20, 21^a^, 21^b^, 21^c^, 21^d^, 22, 23, 24, 25	2490	0.38	0.16	0.60	3.35	0.001
		Delayed	2	10, 17	108	−0.01	−0.61	0.59	−0.04	0.968
Outcome Follow up		No	18	1, 3, 4, 5, 6, 7, 9, 10, 11, 12, 13, 14, 15, 18, 19, 22, 24, 25	2005	0.45	0.22	0.68	3.78	< 0.001
		Yes	10	2, 8, 16, 17, 20, 21^a^, 21^b^, 21^c^, 21^d^, 23	593	0.16	−0.33	0.65	0.63	0.528
Pre-briefing		No	22	1, 2, 3, 6, 7, 8, 9, 12, 13, 14, 16, 17, 18, 19, 20, 21^a^, 21^b^, 21^c^, 21^d^, 22, 23, 24	1862	0.31	0.05	0.57	2.31	0.021
		Yes	6	4, 5, 10, 11, 15, 25	736	0.50	0.13	0.88	2.61	0.009
Debriefing		No	14	7, 11, 12, 13, 16, 18, 19, 20, 21^a^, 21^b^, 21^c^, 21^d^, 22, 23	1169	0.22	−0.10	0.54	1.35	0.178
		Yes	14	1, 2, 3, 4, 5, 6, 8, 9, 10, 14, 15, 17, 24, 25	1429	0.48	0.18	0.78	3.14	0.002
Quality score		< Mean	14	2, 6, 7, 12, 13, 14, 16, 18, 21^a^, 21^b^, 21^c^, 21^d^, 22, 23	1338	0.28	0.02	0.55	2.11	0.035
		≥Mean	14	1, 3, 4, 5, 8, 9, 10, 11, 15, 17, 19, 20, 24, 25	1260	0.43	0.08	0.77	2.40	0.016

Notes. K, number of analysis sets; N, number of participants; CI, confidence interval; IRB, institutional review board; Quasi-E, quasi-experimental study; RCT, randomized controlled trial

Superscripts a, b, c, and d indicate Layton’s (1979) study divided by intervention

Univariate meta-regression analysis was performed to determine the potential impact of study heterogeneity on effect size, which revealed that the following variables had statistically significant effects—specifically, year of publication, IRB-approved studies, the number of participants, study design, intervention type, control group intervention, and intervention time per session (Table 3). The exclusion sensitivity test excluded one study from each of the 28 studies and compared the merged effect size to the original effect size to determine the impact of the estimated effect size [24]. Examining the magnitude and statistical significance of the combined effect sizes of the simulation-based interventions indicated that Hedge’s g was small, ranging from 0.31 to 42, the 95% CI (0.10 to 0.23, 0.52 to 0.61) did not include zero, and all were statistically significant. The effect size was not significantly different from that of Hedge’s g (0.35), including all 28 studies, and all studies were statistically significant. Therefore, the meta-analysis was considered robust (Table 4).

**Table 3:** Meta-regression analysis to evaluate empathy

Covariates (Ref.)	Estimate	SE	95% CI		Z	*p*
			Lower limit	Upper limit		
Year (Ref.: <2019)	0.01	0.00	0.00	0.02	2.25	0.024
IRB (Ref.: No)	0.45	0.09	0.28	0.62	5.25	< 0.001
Research design (Ref.: Quasi-E)	−0.43	0.08	−0.58	−0.27	−5.32	< 0.001
Participants (Ref.: <60)	0.17	0.08	0.01	0.33	2.06	0.040
Intervention type (Ref.: Simulation)	−0.19	0.08	−0.35	−0.03	−2.39	0.017
Control group intervention (Ref.: No or usual)	0.33	0.15	0.04	0.63	2.21	0.027
Intervention time/session (Ref.: not reported or < 1 h)	−0.03	0.09	−0.21	0.15	−0.32	0.746
Intervention duration (Ref.: not reported or < 4weeks)	0.17	0.08	0.01	0.33	2.12	0.034
Outcome measurement time (Ref.: Immediately)	−0.27	0.20	−0.66	0.12	−1.37	0.172
Outcome Follow up (Ref.: No)	−0.16	0.09	−0.34	0.02	−1.70	0.089
Pre-briefing (Ref.: No)	0.12	0.09	−0.05	0.29	1.36	0.173
Debriefing (Ref.: No)	−0.06	0.08	−0.22	0.10	−0.73	0.465
Quality score (Ref.: < mean)	0.14	0.08	−0.02	0.30	1.73	0.083

Notes. Ref., reference; SE, standard error; CI, confidence interval; IRB, institutional review board; Quasi-E, quasi-experimental study

**Table 4:** Exclusion Sensitivity Test for Simulation-Based Interventions

Study ID	K	Hedge’s g	95% CI		Z	*p*
			Lower limit	Upper limit		
1	27	0.36	0.14	0.58	3.22	0.001
2	27	0.34	0.12	0.55	3.07	0.002
3	27	0.35	0.13	0.57	3.13	0.002
4	27	0.36	0.13	0.58	3.08	0.002
5	27	0.32	0.11	0.52	2.96	0.003
6	27	0.37	0.14	0.59	3.24	0.001
7	27	0.34	0.12	0.56	3.05	0.002
8	27	0.31	0.10	0.52	2.94	0.003
9	27	0.35	0.14	0.57	3.17	0.002
10	27	0.36	0.14	0.57	3.19	0.001
11	27	0.37	0.15	0.59	3.27	0.001
12	27	0.36	0.14	0.57	3.22	0.001
13	27	0.34	0.13	0.56	3.11	0.002
14	27	0.38	0.18	0.59	3.65	0.000
15	27	0.36	0.14	0.58	3.17	0.002
16	27	0.37	0.14	0.59	3.24	0.001
17	27	0.38	0.16	0.59	3.43	0.001
18	27	0.37	0.15	0.58	3.29	0.001
19	27	0.33	0.12	0.54	3.02	0.003
20	27	0.42	0.23	0.61	4.26	0.000
21^a^	27	0.36	0.14	0.58	3.26	0.001
21^b^	27	0.34	0.13	0.56	3.10	0.002
21^c^	27	0.37	0.16	0.59	3.41	0.001
21^d^	27	0.36	0.14	0.58	3.27	0.001
22	27	0.33	0.12	0.54	3.03	0.002
23	27	0.36	0.13	0.58	3.11	0.002
24	27	0.33	0.12	0.54	3.03	0.002
25	27	0.33	0.11	0.54	3.00	0.003

Notes. K: number of analysis sets; CI: confidence interval

Superscripts a, b, c, and d indicate Layton’s (1979) study divided by intervention

### Effect of an intervention program on secondary outcomes

Secondary outcomes were empathic communication, interpersonal relationships, and competency, all of which were statistically significant. After the program, empathic communication with Hedge’s g = 1.35 (95% CI:0.25, 2.45), interpersonal relationship with Hedge’s g = 0.52 (95% CI:0.21, 0.84), and competency with Hedge’s g = 0.75 (95% CI:0.24, 1.26), indicating medium to large effect sizes (Table 5).

**Table 5:** Effects of Simulation-Based Interventions on Other Variables

Variables	K	Study ID	*N*	Hedge’s g	95% CI		Z	*p*
					Lower limit	Upper limit		
Empathic communication	6	4, 5, 7, 17, 24, 25	595	1.35	0.25	2.45	2.40	0.016
Interpersonal relationship	9	4, 5, 8, 9, 16, 21^a^, 21^b^, 21^c^, 21^d^	606	0.52	0.21	0.84	3.25	0.001
Competency	9	9, 11, 12, 15, 16, 21^a^, 21^b^, 21^c^, 21^d^	591	0.75	0.24	1.26	2.90	0.004

Notes. K: number of analysis sets; N: number of participants; ES: effect size; CI: confidence interval

Superscripts a, b, c, and d indicate Layton’s (1979) study divided by intervention

### Publication bias

**Fig. 3:**
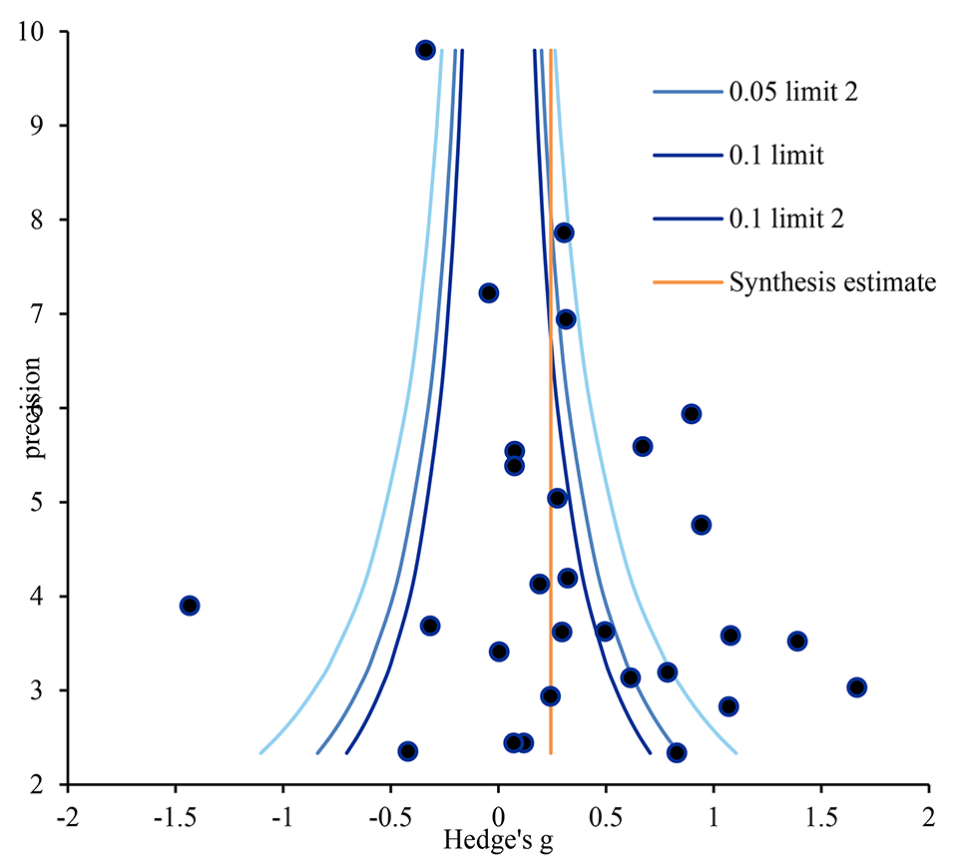
Funnel plot of simulation-based interventions for empathy. Notes. Precision = 1/standard error, 0.05; limit line = 95% confidence limit

Funnel plot analysis was conducted to assess publication bias, which revealed that the individual effect sizes (blue circles) of the 28 included studies were skewed to the right, indicating some degree of publication bias (Fig. 3). For further analysis of publication bias, using the trim-and-fill method, the number of articles that should be added to the study was identified as nine [27]. The corrected effect size of the 37 articles was 0.04 (95%CI: -0.19, 0.26). The effect size of empathy was smaller after correction than before, but the difference was not statistically significant after correction. Moreover, the results of different methods used to detect publication bias differed. Nonetheless, the results obtained using the trim-and-fill method, which is particularly effective in illustrating publication bias in continuous variables, indicated publication bias in this study (Appendix 2).

## Discussion

A random-effects on the results of 28 studies was performed to quantify the influence of simulation on empathy among undergraduate nursing students. The impact of the simulation-based program on empathy showed a small effect size, specifically with an effect size of 0.35. Despite variance within studies and heterogeneity in effects between studies, it was observed that the vast majority of nursing students agree that simulation increases empathy and that empathy is greater after simulation than before. The high I2 indicates significant heterogeneity, which consequently reduces the precision of summary estimates.

This aligns with previous primary research, indicating that to empathize with others beyond oneself, it’s essential to understand the other person’s perspective or position. Moreover, research suggests that such empathy can be cultivated through education [28]. This finding is also consistent with a previous study reporting that learning could improve empathy and a meta-analysis finding that empathy training improved empathy [17, 29, 30]. This study corroborates earlier primary research findings suggesting that empathy training ought to incorporate real-life experiences via imagination and simulations, with a focus on understanding the unobservable mental processes of others [31].

Based on a meta-regression analysis evaluating empathy [17], the factors influencing improvements in empathy are discussed below. Initially, upon scrutinizing the content of recent simulations (since 2019), they delineate as follows: Publication years after 2019 had a more significant impact on empathy than publication years before 2019. The COVID-19 pandemic has significantly influenced prelicensure nursing education, resulting in extensive disruptions that potentially affect the learning and engagement outcomes of nursing students [31]. These results reflect the diversification and sophistication of simulation education. This is because, reportedly, nursing schools in Korea have been educating and evaluating core nursing skills designated by the Korean Accreditation Board of Nursing Education as curricular and extracurricular programs to improve the clinical performance of nursing students, with an increasing number of simulation classes based on clinical scenarios similar to the clinical environment since the 2000s [32]. Since 2019, simulations have been conducted systematically and actively. Thus, the impact on empathy was significantly greater after 2019.

The causes of heterogeneity in characteristics are as follows: The effect on empathy was notably stronger with IRB approval, implying that undergoing an IRB review may signal a scientifically and ethically robust study design. Ensuring scientifically sound design and impact evaluation is crucial, even with the same program. Concerning study design, empathy’s impact was more pronounced in quasi-experimental studies compared to randomized controlled trials (RCTs). Rigorous designs, as seen in certain RCTs with multiple controls, might lead to conservative estimates of simulation effects on empathy due to tight control. Conversely, quasi-experimental studies conducted in natural learning environments suggest empathy impacts may stem from factors beyond simulation. However, further validation through research is needed. Moreover, empathy’s impact was significantly higher with 60 or more participants, likely due to increased effect power. Hence, repeated studies with sufficient participant numbers are essential for evaluating empathy improvements.

By program type, scenario-based simulations had a more significant impact on empathy than role-playing, which is in line with a previous study suggesting that role-play is usually based on a simple situation [33]. By contrast, the simulation was based on a structured scenario that allowed participants to indirectly experience the care recipient’s condition, thereby matching another person’s mind with their mental state. Moreover, role-playing has been found to contribute to empathy, as reported in a previous study in which nursing students’ critical thinking and emotional intelligence increased significantly after learning digital storytelling problem-based learning through role-playing, and a case study containing the care recipient’s disease experience and overall clinical situation [34]. More elaborate settings, assumptions, and preparations for the situation are needed to enable students to experience what being in the situation feels like rather than merely playing a role, which is expected to allow students to be more immersed cognitively and emotionally engaged with the target situation.

The intervention duration was significantly longer for four weeks or more than four weeks than for non-reported or less than four weeks, suggesting that the intervention should be at least four weeks in line with the idea that empathy is formed through continuous and steady learning [1]. This finding indicates that empathy cannot be improved through a short period of experience or training. Instead, empathy, as a process of integrating experiences and existing perceptions, is formed over time.

Other variables whose effects on empathy were not statistically significant were as follows: There were no significant differences in the time per intervention session, whether the outcome measurement time was immediate or delayed, outcome follow-up, prebriefing, debriefing, or quality score. In typical simulation training, prebriefing and debriefing are considered essential and reflective. Nevertheless, this study found no significant effect of pre-or debriefing on empathy, suggesting that the simulation context in which empathy is provided is essential, considering the nature of empathy. However, further studies on this topic are required. Furthermore, in this study, empathy was assessed using a variety of measurement tools. We also recommend that future analyses take into account the specific measurement tools employed.

The findings of this study affirm that simulation-based education, when employed across diverse clinical contexts such as women’s health, operating room scenarios, psychiatric nursing, and geriatric nursing, constitutes a fundamental approach for fostering empathy among nursing students. Among the myriad approaches aimed at enhancing empathy among medical students, the implementation of “patient simulation”—involving students in a curriculum that mirrors real patient encounters—has been noted as effective [35]. Furthermore, previous studies examining the relationship between proficiency and person-centered care competence have consistently demonstrated a positive correlation between empathy and competence in delivering person-centered care [36, 37].

## Conclusion

In this study, we conducted a meta-analysis of research exploring the impact of simulation-based education on empathy. Our findings indicate that simulation-based training across diverse scenarios can indeed enhance empathy levels. Specifically, focusing on immersive simulations conducted for a minimum duration of four weeks, spanning a range of authentic clinical contexts, proved to be particularly effective. Moreover, our study underscores the holistic nature of empathy, revealing its interconnectedness with other nursing competencies. As such, further research in this domain is warranted to deepen our understanding and refine instructional methodologies.

### Electronic supplementary material

Below is the link to the electronic supplementary material.

Supplementary Material 1

## Data Availability

﻿The datasets used and/or analysed during the current study are available from the corresponding author on reasonable request.

## Abbreviations

PICO-SD: Population, Intervention, Comparison, Outcome, and Study Design
PRISMA: Preferred Reporting Items for Systematic Reviews and Meta-Analyses
SD: Study Design
RCTs: Randomized Controlled Trials
RISS: Research Information Sharing Service
PROSPERO: Prospective Register of Systematic Reviews
MeSH: Medical Subject Headings
JBI: Joanna Briggs Institute
JSE-HP-S: Jefferson Scale of Empathy-Health Profession-Student
