# Supplementary material for: Effectiveness of simulation-based interventions on empathy enhancement among nursing students: a systematic literature review and meta-analysis
Source: BMC Nurs. 2024 May 11;23:319. doi: 10.1186/s12912-024-01944-7 (PMC11088026; doi:10.1186/s12912-024-01944-7)
Supplement: Supplementary file 1 — Supplementary Material 1 [file 12912_2024_1944_MOESM1_ESM.docx]

**Appendix 1.** Descriptive summary of the included studies.

| Study ID | Author (year) | IRB | Participants | Research design | Intervention type | Control G intervention | Intervention duration | Intervention session | Session time | Measurement time | Delayed Measurement | Outcome variable | Empathy scale |
| --- | --- | --- | --- | --- | --- | --- | --- | --- | --- | --- | --- | --- | --- |
| 1 | Slagle (2019) | Yes | 76  Associate degree nursing students  (E: 26, C: 50) | Quasi | Simulation | Conventional | Not  reported | Not reported | Not reported | Immediately | None | Emotional intelligence | Train Emotional Intelligence Questionnaire: Short Form (TEIQue-SF) |
| 2 | Choi et al. (2016) | Yes | 44 Undergraduate nursing students  (E: 22, C: 22) | Quasi | Simulation | Comparison (clinical practicum) | 5weeks | Not reported | 30 min | Immediately | Yes | Empathy,  Clinical questions, Mental illness prejudice,  Self-efficacy and satisfaction | Empathy Construct Rating Scale developed by Monica(1981) |
| 3 | Fernandez-Gutierrez et al. (2022) | No | 55  3rd year nursing students (E:24, C:31) | RCT | Simulation | Conventional | 16weeks | 12  sessions | 5 hours | Immediately | None | Empathy,  Attitude | Spanish adaptation of the Jefferson Scale of Empathy, student version (JES-S) |
| 4 | Ding et al. (2020) | Yes | 250  Nursing students in clinical internship  (E: 125,  C: 125) | Quasi | Simulation | Conventional | 5 days | 3sessions | 5 days | Immediately | None | Empathy,  Communication competence,  Professional identity | The Jefferson Scale of Empathy-Health Profession-Student(JSE-HP-S) |
| 5 | Sung et al. (2022) | Yes | 62  First-grade nursing students (E:32, C:30) | Quasi | Role-play | Comparison (portable mini cards containing the principles of NVC) | 6weeks | 6  sessions | 120 min | Immediately | None | Empathy,  Self-esteem,  Interpersonal relationship,  Communication competency | the empathy scale, originally developed by Davis(1983) and Bryant(1982) |
| 6 | Riess (2018) | Yes | 123 Nursing students (E:59, C:64) | Quasi | Simulation | Conventional | Not reported | Not reported | Not reported | Immediately | None | Empathy,  Self-confidence,  Satisfaction | Jefferson Scale of Empathy-Health Professions Student |
| 7 | Li et al. (2019) | Yes | 132  1st-year undergraduate nursing students (E:66, C:66) | RCT | Simulation | Conventional | 8 weeks | 16sessions | 30 min | Immediately | None | Empathy,  Communication,  Self-efficacy | Jefferson Scale of Empathy-Health Professionals |
| 8 | Haley et al. (2017) | Yes | 50  Nursing students (E:26, C:24) | RCT | Simulation | Not reported | Not reported | Not reported | 20 min | Immediately | Yes | Empathy,  Active listening,  Self-awareness,  Patient center care (PCC) | The Kiersma-Chen Empathy Scale |
| 9 | Huang et al. (2023) | Yes | 72  1st-year nursing students (E:40, C:32) | Quasi | Role-play | Conventional | 6 weeks | 6  sessions | 50 min | Immediately | None | Empathy,  Caring behavior,  Competence | the Jefferson Scale of Empathy-Healthcare Providers (JSE-HP) |
| 10 | Shin et al. (2023) | Yes | 53  3rd or 4th year undergrduate nursing students (E:26, C:27) | Quasi | Simulation | Conventional | Not reported | Not  reported | 45 min | Delayed (3 days after the program) | None | Empathy,  Cultural competency | Interpersonal Reactivity Index(IRI) developed by Davis(1980) |
| 11 | Chen et al. (2020) | No | 209  1st year nursing students (E:99, C:110) | RCT | Simulation | Conventional | 8weeks | Not reported | Not reported | Immediately | None | Empathy,  Knowledge,  Confidence,  Intention to learn,  Health assessment skills | Toronto Empathy Questionnaire(TEQ) |
| 12 | Ma et al. (2021) | Yes | 35 Undergraduate nursing students (E:19, C:16) | RCT | Role-play | Comparison (Between-subject design) | Not reported | Not reported | 10 min | Immediately | None | Empathy,  Spatial presence | Scale developed by Campbell and Babrow(2004) |
| 13 | Lobchuk et al. (2018) | Yes | 42 Undergraduate students (E:24, C:18) | RCT | Simulation | Comparison (Video-feedback only | 4 weeks | Not reported | Not reported | Immediately | None | Empathy,  Health risk behavior,  Carers to indicate the health risk behavior,  Student perceptual understanding of carers' thoughts and feelings experienced during the dialogue | Consultation and Relational Empathy (CARE) |
| 14 | Levett-Jones (2017) | No | 390  2nd-year bachelor of nursing students  (E: 202, C:188) | RCT | Role-play | rehabilitation nurse role) | Not reported | Not reported | 1hour | Immediately | None | Empathy | Comprehensive State Empathy Scale (CSES) (Everson et al., under review) |
| 15 | Lee et al. (2018) | Yes | 103  2nd-year BSN students (E:48, C:55) | Quasi | Role-play | Conventional | 1 weeks | Not reported | 10hours +15min | Immediately | None | Empathy | Jefferson Scale of Empathy-Health Profession-Student version (JSE-HP-S) |
| 16 | Bas-Sarmiento et al. (2019) | No | 116  2nd-year nursing students (E:59, C:57) | RCT | Role-play | Conventional | 2 weeks | 7 sessions | 2 hours | Immediately | Yes | Empathy | The Consultation and Relational Empathy Measure  Jefferson Scale of Empathy student version  Reynolds Empathy Scale  Carkhuff Scale |
| 17 | Regan (2000) | Yes | 55  1st or 2nd semester sophomore nursing students (E:28, C:27) | Quasi | Role-play | Conventional | 8 weeks | Not reported | One half hour | Delayed (10th week of semester) | Yes | Empathy | LaMonica Empathy Profile (LEP) |
| 18 | Matteoli (1989) | Yes | 47 Undergraduate nursing students (E:21, C:26) | Quasi | Simulation | Comparison (audiotaped demonstration) | Not reported | 3 sessions | 2 hours | Immediately | None | Empathy  Cognitive style  Empathy communication behavior | Nurse Empathy Checklist (NEC) |
| 19 | Yang et al. (2020) | Yes | 102  4th Undergraduate nursing students (E:57, C:45) | Quasi | Role-play | Comparison (empathy education) | 2 weeks | 4 sessions | 3 hours | Immediately | None | Empathy | Jefferson Scale of Empathy-Health Providers (JSE-HPs |
| 20 | Larti et al. (2018) | Yes | 77 Operating room nursing students (E:36, C:41) | RCT | Role-play | None | 3 weeks | 3 sessions | 4 hours | Immediately | Yes | Empathy | Jefferson Scale of Empathy-Health Profession Student version |
| 21 | Layton (1979) | Yes | 56  Junior and senior baccalaureate nursing students (E^a^:11, E^b^:11, E^c^:10, E^d^:11, C:13) | RCT | Role-play | None | Not reported | Not reported | Not reported | Immediately | Yes | Empathy | Carkhugg Empathic Understanding in Interpersonal Processes Scale |
| 22 | Thomas et al. (2020) | Yes | 158 Nursing students (E: 90, C:68) | Quasi | Role-play | Conventional | Not reported | Not reported | Not reported | Immediately | None | Empathy | Kiersma-Chen Empathy Scale (KCES, Kiersma et al., 2013) |
| 23 | Yu et al. (2021) | Yes | 195  2nd year nursing students (E:98, C:97) | RCT | Simulation | Conventional | Not reported | Not reported | Not reported | Immediately | Yes | Empathy | Jefferson Scale of Empathy (JSE) - Health Professional version |
| 24 | Jeong (2019) | Yes | 37  2nd year nursing students (E:17, C:20) | Quasi | Simulation | Conventional | 4 weeks | 4 sessions | 4 hours | Immediately | None | Empathy  Communicative competence  Self-efficacy | Jefferson scale of empath |
| 25 | Seo et al. (2019) | No | 59  Nursing students (E:29, C:30) | Quasi | Role-play | Conventional | 3 weeks | 6 sessions | total 5-6 hours | Immediately | None | Empathy  Communicative competence | Empathy Scale developed by Davis(1986) |

Notes. IRB: Institutional Review Board; E: experimental group; C: control group; RCT: randomized controlled trial; Quasi: quasi-experimental study; E^a^: modeling group; E^b^: Modeling+Labeling group; E^c^: Modeling+rehearsal group; E^d^: Modeling+Labeling+rehearsal group.

**Appendix 2.** Publication Bias Test of Simulation-Based Interventions on Empathy.

| Begg's test | Tau b | K | S (P-Q) | ties | Z | p |
| --- | --- | --- | --- | --- | --- | --- |
| Standard | 0.11 | 28 | 40 | 0 | 0.79 | 0.429 |
| Corrected | 0.10 | 28 | 40 | 0 | 0.77 | 0.441 |
| Egger's regression test | Coefficient | SE | 95% CI | | Z | p |
|  |  |  | Lower limit | Upper limit |  |  |
| Intercept | 2.10 | 1.22 | −0.29 | 4.49 | 1.72 | 0.085 |
| Slope | −0.17 | 0.26 | −0.67 | 0.34 | −0.65 | 0.517 |
| Trim and fill method | K | Hedge’s g | 95% CI | | Z | p |
|  |  |  | Lower limit | Upper limit |  |  |
| Original | 28 | 0.35 | 0.14 | 0.57 | 3.27 | 0.001 |
| Corrected | 37 | 0.04 | −0.19 | 0.26 | 0.30 | 0.760 |

Notes. Begg's test for rank correlation; Egger's regression test for zero intercept; SE, standard error; CI, confidence interval; K, number of analysis sets.

**Appendix 3. References**

1. Bas-Sarmiento P, Fernández-Gutiérrez M, Díaz-Rodríguez M. Teaching empathy to nursing students: A randomised controlled trial. Nurse Educ Today. 2019;80:40-51. https://doi.org/10.1016/j.nedt.2019.06.002.

2. Chen HC, Ignacio J, Yobas P. Evaluation of the symptom-focused health assessment and empathy program for undergraduate nursing students A randomized controlled trial. Nurse Educ Today. 2020;94:104566. https://doi.org/10.1016/j.nedt.2020.104566.

3. Choi H, Hwang B, Kim S, Ko H, Kim S, Kim C. Clinical Education in psychiatric mental health nursing: Overcoming current challenges. Nurse Educ Today. 2016;39:109–115. http://dx.doi.org/10.1016/j.nedt.2016.01.021.

4. Ding X, Wang L, Sun J, Li DY, Zheng BY, He SW, Zhu LH, Latour JM. Effectiveness of empathy clinical education for children's nursing students: A quasi-experimental study. Nurse Educ Today. 2020;85:104260. https://doi.org/10.1016/j.nedt.2019.104260.

5. Seo DH, Jeong IJ. The Effects of Role Playing on Empathy and Communication Competence for Nursing Students in Psychiatric Mental Health Nursing Practicum. Journal of KOEN. 2019;13(4): 263-270. https://doi.org/10.21184/jkeia.2019.6.13.4.263.

6. Fernández-Gutiérrez M, Bas-Sarmiento P, Del Pino-Chinchilla H, Poza-Méndez M, Marín-Paz AJ. Effectiveness of a multimodal intervention and the simulation flow to improve empathy and attitudes towards older adults in nursing students: A crossover randomised controlled trial. Nurse Educ. Pract. 2022;64:103430. https://doi.org/10.1016/j.nepr.2022.103430.

7. Haley B, Heo S, Wright PB, Barone CP, Rettigantid MR, Anders ME. Effects of Using an Advancing Care Excellence for Seniors Simulation Scenario on Nursing Student Empathy: A Randomized Controlled Trial. Clin. Simul. Nurs. 2017;13:511-519. http://dx.doi.org/10.1016/j.ecns.2017.06.003.

8. Huang SM, Fang SC, Lee SY, Yu PJ, Chen CJ, Lin YS. Effects of video-recorded role-play and guided reflection on nursing student empathy, caring behavior and competence: A two-group pretest-posttest study. Nurse Educ. Pract. 2023;67:103560. https://doi.org/10.1016/j.nepr.2023.103560.

9. Kye-A J. Development and Effects of Simulation-Based Empathy Improvement Program for Nursing students. Kangwon National University. 2019

10. Larti N, Ashouri E, Aarabi A. The effect of an empathy role-playing program for operating room nursing students in Iran. J Educ Eval Health Prof. 2018;15(29). https://doi.org/10.3352/jeehp.2018.15.29.

11. Layton JM. The use of modeling to teach empathy to nursing students. Res Nurs Health. 1979;2:163-176. https://doi.org/10.1002/nur.4770020405.

12. Lee KC, Yu CC, Hsieh PL, Li CC, Chao YC. Situated teaching improves empathy learning of the students in a BSN program: A quasi-experimental study. Nurse Educ Today. 2018;64:138-143. https://doi.org/10.1016/j.nedt.2018.02.013.

13. Levett-Jones T, Lapkin S, Govind N, Pich J, Hoffman K, Jeong SY, Norton CA, Noble D, Maclellan L, Robinson-Reilly M, Everson N. Measuring the impact of a 'point of view' disability simulation on nursing students' empathy using the Comprehensive State Empathy Scale. Nurse Educ Today. 2017;59:75-81. http://dx.doi.org/10.1016/j.nedt.2017.09.007.

14. Li J, Li X, Gu L, Zhang R, Zhao R, Cai Q, Lu Y, Wang H, Meng Q, Wei H. Effects of Simulation-Based Deliberate Practice on Nursing Students' Communication, Empathy, and Self-Efficacy. J Nurs Educ. 2019;58(12):681-689. https://doi.org/10.3928/01484834-20191120-02.

15. Lobchuk M, Hoplock L, Halas G, West C, Dika C, Schroeder W, Ashcroft T, Clouston KC, Lemoine J. Heart health whispering A randomized, controlled pilot study to promote nursing student perspective-taking on carers' health risk behaviors. BMC Nursing. 2018;17(21). https://doi.org/10.1186/s12912-018-0291-1.

16. Ma Z, Huang KT, Yao L. Feasibility of a Computer Role-Playing Game to Promote Empathy in Nursing Students The Role of Immersiveness and Perspective. Cyberpsychol Behav Soc Netw. 2021;24(11):750-755. https://doi.org/10.1089/cyber.2020.0371.

17. Matteoli R. The effect of modeling instruction and baccalaureate nursing student cognitive style on expressed empathy as measured by the Nurse Empathy Checklist. University of San Francisco. 1989

18. Regan R. The effect of gaming on the empathic communication of associate degree nursing students. Widener University. 2020

19. Riess DL. Effects of Simulated Clinical Experiences on Empathy, Self-Confidence, and Satisfaction in Nursing Students. Walden University. 2018

20. Shin M, Na H, Kim S. Enhancing cultural competency and empathy toward foreign patients for Korean nursing students through a simulation: A quasi-experimental study. Nurse Educ. Pract. 2023;69. https://doi.org/10.1016/j.nepr.2023.103615.

21. Slagle T. Advancing Emotional Intelligence in Nursing Students through Debriefing. Capella University. 2019

22. Sung J, Kweon Y. Effects of a Nonviolent Communication-Based Empathy Education Program for Nursing Students: A Quasi-Experimental Pilot Study. Nurs. Rep. 2022;12: 824–835. https://doi.org/10.3390/nursrep12040080.

23. Thomas V. Using Simulation Exercises to Teach Nursing Students the Concept of Empathy. ABNF J. 2020;31(2):61-66.

24. Yang C, Zhu YL, Xia BY, Li YW, Zhang J. The effect of structured empathy education on empathy competency of undergraduate nursing interns: A quasi-experimental study. Nurse Educ Today. 2020;85. https://doi.org/10.1016/j.nedt.2019.104296.

25. Yu J, Parsons GS, Lancastle D, Tonkin ET, Ganesh S. Walking in Their Shoes The effects of an immersive digital story intervention on empathy in nursing students. Nurs. Open. 2021;8:2813–2823. https://doi.org/10.1002/nop2.860.
